# Association of Alzheimer's disease concerns with amyloid burden and lifestyle behaviors in cognitively unimpaired older adults

**DOI:** 10.1002/alz.70225

**Published:** 2025-06-06

**Authors:** Francesca R. Farina, Marc Bennett, Joshua D. Grill, Reisa Sperling, Brian Lawlor, James W. Griffith

**Affiliations:** ^1^ Pritzker School of Medicine The University of Chicago Chicago Illinois USA; ^2^ Global Brain Health Institute Trinity College Dublin Dublin Ireland; ^3^ Department of Psychology John Henry Newman Building University College Dublin Dublin Ireland; ^4^ Institute for Memory Impairments and Neurological Disorders University of California Irvine Irvine California USA; ^5^ Center for Alzheimer Research and Treatment Brigham and Women's Hospital, Massachusetts General Hospital, Harvard Medical School Boston Massachusetts USA

**Keywords:** Alzheimer's disease concerns, amyloid burden, *APOE* ε4, cognitively unimpaired, lifestyle behaviors

## Abstract

**INTRODUCTION:**

The extent to which Alzheimer's disease (AD) concerns relate to pathological changes in cognitively unimpaired populations is unclear.

**METHODS:**

We analyzed screening data from the Anti‐Amyloid Treatment in Asymptomatic Alzheimer's (A4) study to determine if AD concerns are associated with amyloid burden in cognitively unimpaired older adults, and how they relate to lifestyle. AD concerns were measured using the six‐item Concerns about Alzheimer's Disease Questionnaire. Regression estimated the association of AD concerns with amyloid burden, adjusting for covariates.

**RESULTS:**

Of 4460 individuals, AD concerns were elevated in women, people with a dementia family history, apolipoprotein E (*APOE*) ε4 carriers, and individuals who did not meet walking or sleep guidelines. AD concerns were associated with higher amyloid burden (*β* [95% CI] = 0.002 [0.001–0.003], *p* = 0.007), with stronger effects in *APOE* ε4 carriers.

**DISCUSSION:**

AD concerns were associated with a core diagnostic AD biomarker. Assessing AD concerns could inform future recruitment strategies.

Clinical Registration Trial:: ClinicalTrials.gov: ID NCT02008357.

**Highlights:**

- Alzheimer's disease (AD) concerns were associated with higher amyloid burden.
- AD concerns were associated with higher depressive symptoms in apolipoprotein E (*APOE*) ε4 carriers.
- Concerns were associated with a lower likelihood of meeting daily walking guidelines.
- Associations were stronger in *APOE* ε4 carriers compared to non‐carriers.

## 1 BACKGROUND

In June 2024, the Alzheimer's Association Workgroup published new diagnostic criteria for Alzheimer's disease (AD), which for the first time, defined the disease based on biological changes rather than clinical symptoms.^1^ Using these criteria, AD could be diagnosed by abnormality of core biomarkers such as amyloid positron emission tomography (PET). Use of biomarkers to diagnose AD in asymptomatic individuals is widely discouraged.^2^ However, this may change as new treatments demonstrate efficacy in preclinical disease stages.

Concerns about developing AD are common, particularly among older adults and those with a family history of dementia.^3^, ^4^, ^5^ Although there is no consensus definition or measurement, AD concerns can refer to an individual's level of concern and perceived probability of developing AD.^6^ AD concerns are distinct from “dementia worry,” in that AD concerns focus specifically on AD, as opposed to the umbrella term “dementia.” Dementia worry is also conceptually broader, encompassing a range from occasional concerns about forgetfulness to preoccupation with dementia that impairs functioning.^7^

Multiple studies have reported associations of AD concerns with depression, anxiety, and subjective cognitive decline (SCD).^7^, ^8^, ^9^ In individuals with SCD, having AD concerns has been shown to indicate pathological changes, including amyloid accumulation.^10^ However, the association of AD concerns with such pathological changes in cognitively unimpaired individuals has not been well established. One recent study reported higher AD concerns among cognitively unimpaired individuals with higher amyloid levels prior to biomarker disclosure.^11^ A limitation, however, was that the study included only individuals willing to learn their amyloid imaging results, which may have biased the sample. In addition, the analysis did not account for key demographic, psychological, or cognitive factors that could influence the association between AD concerns and amyloid burden, particularly anxiety and depression. Thus, the extent to which AD concerns uniquely relate to core biomarkers like amyloid in the preclinical stage of the disease remains unclear.

Building on previous work, we analyzed secondary data from a large cohort of cognitively unimpaired older adults screened for the Anti‐Amyloid Treatment in Asymptomatic Alzheimer's (A4) Study multicenter prevention trial^12^ to assess whether concerns about developing AD are associated with amyloid burden after adjusting for anxiety and depressive symptoms, subjective and performance‐based cognition, dementia family history, and genetic risk. We also investigated associations of AD concerns with psychological well‐being and healthy lifestyle behaviors. Our rationale followed the framework of fear‐avoidance models,^13^, ^14^ whereby AD concerns may contribute to avoidance behaviors (e.g., withdrawal from daily activities like physical activity or increased alcohol use) or mood changes (e.g., depressive symptoms).

## 2 METHODS

### 2.1 Study

Participants were cognitively unimpaired older adults (65–85 years of age) who were screened to assess eligibility for the A4 Study, a secondary AD prevention trial.^15^ Cognitive inclusion criteria were Clinical Dementia Rating (CDR) of 0, Mini‐Mental State Examination (MMSE) score greater than or equal to 25, and Logical Memory II subscale delayed recall of the Wechsler Memory Scale Revised score of 6–18.^16^, ^17^ Data for the A4 Study were collected from April 2014 to December 2017, across 67 sites in the United States, Canada, Australia, and Japan. The study protocol was approved by institutional review boards (IRBs) at each study site, and all participants provided written informed consent. The study was performed in accordance with the ethical standards as laid down in the 1964 Declaration of Helsinki and its later amendments. Diversity, equity, and inclusion (DEI) considerations in the A4 Study have been reported in detail previously.^18^, ^19^ The trial team used a multitude of recruitment strategies to promote inclusion of under‐represented groups, including direct site investigator clinical practices, outside physician referrals, organization referrals (e.g., Alzheimer's Association), local community outreach, paid advertisements, and earned media (e.g., news or other non‐paid content). In our analyses, we specifically considered the representation of key demographic characteristics (i.e., sex, race, and ethnicity).

For the current study, we accessed publicly available de‐identified screening data; thus, the study was exempt from IRB review per the Common Rule. Data were obtained from the A4 Study website (www.a4studydata.org) and last analyzed in September 2024. Secondary data used in the current study include demographic information, F‐florbetapir amyloid‐beta (Aβ) PET at baseline, apolipoprotein E (*APOE*) genotype, participant‐reported parental history of dementia (i.e., maternal and paternal), state anxiety scores from the State‐Trait Anxiety Inventory (STAI), Geriatric Depression Scale (GDS) scores, MMSE scores, Cognitive Function Index (CFI)^20^ scores, Concerns about Alzheimer's Disease Questionnaire (CADQ) scores,^11^ and lifestyle behaviors (i.e., aerobic exercise, walking, sleep, smoking status, and alcohol). Lifestyle behaviors were measured using a self‐reported lifestyle habits questionnaire, which included average (1) number of hours of aerobic exercise (i.e., jogging, swimming, cycling) per week, (2) number of minutes of walking per week, (3) hours of sleep per night, (4) number of packs of smoked per day, and (5) number of alcohol drinks consumed per day. Publicly available data for smoking and alcohol were dichotomized into current users versus non‐users. Lifestyle variables were chosen based on associations with dementia risk,^21^, ^22^ and practical availability in the dataset.

The CADQ is a six‐item questionnaire assessing level of concern about developing AD dementia (e.g., “My concern about developing Alzheimer's disease dementia is greater than my concern about other medical problems”) and perceived probability of developing AD (e.g., “I believe that I will someday develop Alzheimer's disease dementia”) adapted from previous literature.^6^ Participants indicate their level of agreement with each item on a 5‐point scale ranging from Strongly Disagree (1) to Strongly Agree (5). In this way, higher scores indicate a greater level of concern about AD. Scores range from 6–30. All individual CADQ items are presented in Table S1.

Cognitive testing and questionnaires were administered before amyloid PET and *APOE* genotyping, thereby eliminating any potential bias associated with participant knowledge of amyloid or *APOE* status. We used the previously published Aβ‐PET cortical composite standardized uptake value ratio (SUVR) with whole cerebellum reference as a continuous measure of Aβ burden.^16^ We followed the Strengthening the Reporting of Observational Studies in Epidemiology (STROBE) reporting guideline for a cross‐sectional study.

### 2.2 Statistical analyses

Statistical analyses were performed using R version 4.4.1. To check the assumptions of our statistical analyses, we conducted visual inspection of scatterplots and Q–Q plots to test for linearity and normality, respectively. We also conducted Shapiro–Wilk tests (*p* > 0.05) for normality. For the regression models, we used variance inflation factors (VIFs) to test for multicollinearity with an accepted threshold of 5. VIFs above 5 were investigated and followed up with additional appropriate analyses (e.g., splitting the dataset by group to simplify models and avoid multicollinearity). We used two‐sided Wilcoxon and Kruskal–Wallis tests to compare mean concerns about developing AD (CADQ) scores across demographic (i.e., sex, race, and ethnicity), family history, *APOE* ε4 carrier status, and depression, and lifestyle groups based on alcohol use, smoking, aerobic exercise, walking, and sleep. The publicly available data for alcohol use and smoking were dichotomous (yes/no). We assessed aerobic exercise, walking, and sleep using the Centers for Disease Control and Prevention (CDC) recommended guidelines for older adults. Aerobic exercise was dichotomized as 2.5 h or more per week versus less than 2.5 h per week. Walking was dichotomized as 30 min or more per day versus less than 30 min per day. Sleep was dichotomized as 7 h or more per night versus less than 7 h per night. Analyses were followed up with logistic regression models to adjust for potential cofounders (age, sex, education, anxiety, and depression). We also conducted descriptive analysis to assess the concordance between parental history of dementia and *APOE* ε4 carrier status.

**RESEARCH IN CONTEXT**

1. **Systematic review**: The authors reviewed the literature using traditional sources. Few studies have investigated the association of Alzheimer's disease (AD) concerns with pathological changes in cognitively unimpaired populations. We analyzed the association between AD concerns and amyloid burden in cognitively unimpaired older adults, adjusting for dementia family history, genetic risk, cognition, anxiety, and depressive symptoms. We also investigated how AD concerns relate to modifiable lifestyle behaviors.
2. **Interpretation**: Our findings showed that AD concerns are associated with elevated amyloid burden and with stronger effects in apolipoprotein E (*APOE*) ε4 carriers. AD concerns were also associated with higher depressive symptoms and a lower likelihood of meeting daily walking guidelines.
3. **Future directions**: Future large studies in more diverse samples are required to better understand the association of AD concerns with AD pathology over time, and to assess the utility of AD concerns as a screening tool for lifestyle interventions.

We then used linear regression models to assess the association between concerns about developing AD with Aβ‐PET covarying for demographics (age, sex, and years of education), state anxiety, depression, MMSE score, self‐reported cognition (CFI score), dementia family history, and *APOE* ε4 status. To determine the unique value of AD concerns above and beyond covariates, we compared two models: one with all variables except CADQ scores (Model 1) and one with all variables including CADQ scores (Model 2). We statistically compared Models 1 and 2 using analysis of variance (ANOVA). We also assessed interactions between (1) sex and AD concerns, (2) *APOE* ε4 status and AD concerns, and (3) family history of dementia and AD concerns in Aβ‐PET, using separate models. Significant models were followed up with subgroup analyses. Model 3 was compared to the best‐performing main‐effects model. Finally, we examined the predictive power of individual items on the CADQ using the same covariates as in Model 2.

## 3 RESULTS

### 3.1 Association of AD concerns with demographics and lifestyle factors

**FIGURE 1:**
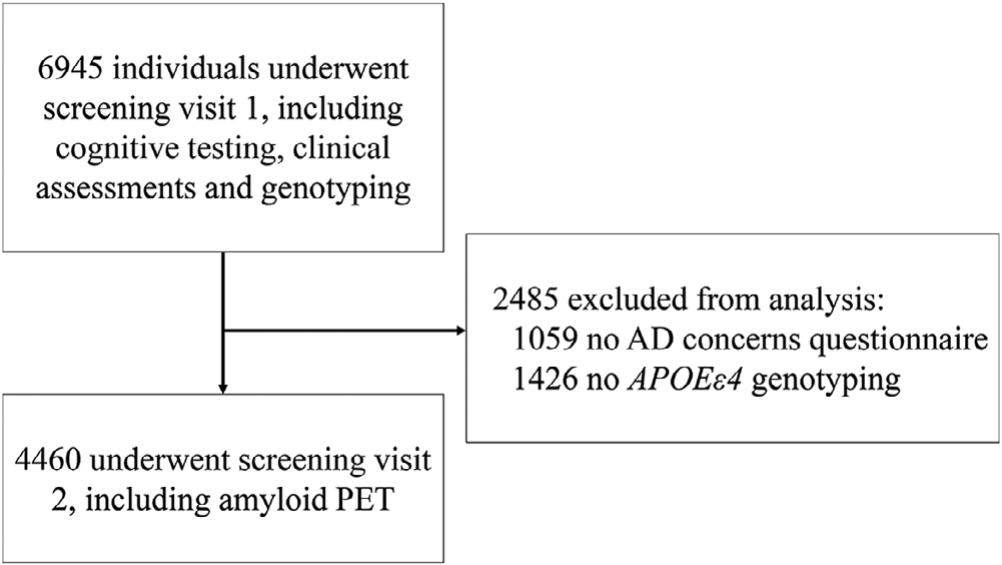
Participant flowchart showing population included in the analysis.

In total, 4460 participants were included in the analysis (see Figure 1). Participant characteristics are presented in Table 1. Of those participants who were *APOE ε*4 carriers, 142 (9.1%) were homozygotes and 1421 (90.9%) were heterozygotes (116 ε2/ε4 and 1305 ε3/ε4). Mean CADQ scores were significantly higher among: (1) female compared to male participants, (2) Latino compared to non‐Latino participants, (3) individuals with a parental family history of dementia compared to those without, and (4) individuals who were later found to be *APOE* ε4 carriers compared to non‐carriers. Among *APOE* ε4 carriers, no significant difference in AD concerns was found between homozygotes and heterozygotes (22.2 [4.4] vs 21.5 [4.6], *p* = 0.011).

**TABLE 1:** Participant demographic characteristics.

Characteristic	Total	CADQ, mean (SD)	*p*‐value
Age, mean (SD), y	71.29 (4.67)	–	–
Education, mean (SD), y	16.58 (2.83)	–	–
Sex, no. (%)			**<0.001**
Female	2649 (59.39)	21.3 (4.62)	
Male	1811 (40.61)	20.34 (4.64)	
Race, no. (%)			–
Asian	169 (3.79)	20.47 (4.91)	
American Indian or Alaska Native	9 (0.20)	18.44 (7.35)	
Black or African American	159 (3.57)	20.62 (4.86)	
Native Hawaiian or Other Pacific Islander	2 (0.05)	17.5 (0.71)	
White	4068 (91.21)	20.94 (4.63)	
Unknown	126 (1.19)	21.53 (4.85)	
Ethnicity, no. (%)			**0.019**
Latino	140 (3.14)	21.76 (4.99)	
Not Latino	4284 (96.05)	20.88 (4.64)	
Unknown	36 (0.81)	21.47 (4.5)	
Family history of dementia, no. (%)			**<0.001**
Yes	2876 (64.48)	21.71 (4.36)	
No	1584 (35.52)	19.46 (4.83)	
*APOE* ε4 carrier, no. (%)			**<0.001**
Yes	1563 (35.05)	21.56 (4.54)	
No	2897 (64.96)	20.56 (4.68)	
MMSE, mean (SD)	28.81 (1.21)	–	–
CFI, mean (SD)	2.03 (2.08)	–	–
PET SUVR, mean (SD)	1.09 (0.19)	–	–
Current alcohol consumer, no. (%)			0.352
Yes	2215 (49.69)	20.84 (4.6)	
No	2243 (50.31)	20.98 (4.71)	
Current smoker, no. (%)			0.482
Yes	75 (1.68)	20.91 (4.65)	
No	4382 (98.32)	21.27 (4.94)	
Aerobic exercise per week, no. (%)			0.544
2.5 h or more	2302 (51.63)	20.87 (4.63)	
Less than 2.5 h	2157 (48.37)	20.96 (4.68)	
Walking per day, no. (%)			**0.032**
30 min or more	3338 (74.91)	20.83 (4.66)	
Less than 30 min	1118 (25.09)	21.16 (4.64)	
Hours of sleep per night, no. (%)			**0.039**
7 h or more	3266 (73.23)	20.83 (4.59)	
Less than 7 h	1194 (26.77)	21.14 (4.81)	
STAI state score, mean (SD)	9.95 (3.12)	–	
GDS score, no. (%)			**<0.001**
0–5 ("normal")	4316 (96.77)	20.86 (4.64)	
5 or greater ("depression")	114 (3.23)	22.49 (4.97)	

*Note*: Participants were allowed to select more than one race. Comparisons across individual participant categories and CADQ scores were done using a Wilcoxon's test with a Holm adjustment to the *p‐*value to account for multiple comparisons.

Abbreviations: *APOE*, apolipoprotein E; CADQ, Concerns about developing Alzheimer's disease Questionnaire; CFI, Cognitive Function Index; MMSE, Mini‐Mental State Examination PET, positron emission tomography; SUVR, standardized uptake value ratio.

Bold text indicates *p* values < 0.05 (i.e., considered statistically significant).

Mean CADQ scores were also higher among participants with GDS scores indicative of depression (vs “normal” range), and those who did not meet CDC recommended daily guidelines for walking (i.e., < 30 min per day) or sleep (i.e., <7 h) compared to those who did meet these criteria. We were unable to compare AD concerns across race categories due to small numbers in some groups, which could lead to potential instability of estimates. No differences were observed for alcohol use, smoking, or aerobic exercise. Follow‐up logistic regression analyses adjusted for confounders showed a significant interaction between AD concerns and *APOE* ε4 for depression (see Table S2). The VIF exceeded 5, so we computed separate regressions for *APOE* ε4 carriers and non‐carriers; higher CADQ scores were significantly positively associated with likelihood of depressive symptoms in *APOE* ε4 carriers (*p* = 0.001), but not in non‐carriers (*p* = 0.278). For walking, higher CADQ scores were negatively associated with the likelihood of meeting daily guidelines after adjusting for confounders (Table S2). The VIFs for *APOE* ε4 and its interaction with AD concerns exceeded 5, so we computed separate regressions for carriers and non‐carriers; higher CADQ scores were significantly positively associated with likelihood of daily guidelines in *APOE* ε4 non‐carriers (*p* = 0.024), but not in carriers (*p* = 0.778). There was a significant interaction between AD concerns and *APOE* ε4 for alcohol use (see Table S2; VIF >5). Separate regressions for *APOE* ε4 carriers and non‐carriers indicated that higher CADQ scores were significantly positively associated with likelihood of being an alcohol consumer in *APOE* ε4 carriers (*p* = 0.047), but not in non‐carriers (*p* = 0.377). The association between CADQ scores and aerobic exercise, smoking, and sleep were not significant after controlling for confounders (Table S2) or after repeating the analyses for *APOE* ε4 carriers and non‐carriers separately.

### 3.2 Association of AD concerns with amyloid burden

Model 1 (covariates only) indicated that older age, female sex, *APOE* ε4 carrier status, parental history of dementia, lower GDS score, lower MMSE score, and higher CFI score (i.e., more cognitive complaints) were all significantly associated with elevated amyloid burden (Table 2). The addition of AD concerns in Model 2 was significant; that is, CADQ scores were significantly associated with elevated amyloid. ANOVA comparing these models indicated that Model 2 was superior to Model 1 (*F*(1, 4442) = 5.55, *p* = 0.01). This indicates that AD concerns were independently associated with amyloid burden, distinct from broader anxiety and depression symptoms, cognitive performance, family history, and genetic risk. Placed in the context of a strong predictor like age, each 1‐unit increase of AD concern was associated with an increase in amyloid burden equivalent to ≈0.23 additional years of age (or 2.7 months).

**TABLE 2:** Amyloid burden linear regression analyses.

	*β*	*β* (SE) (95% CI)	*t*‐value	*p*‐value
**Model 1: Covariates**				
Age	0.168	0.007 (0.001) (0.006 to 0.008)	11.68	**<0.001**
Sex	−0.034	−0.01 (0.01) (−0.03 to −0.003)	−2.42	**0.02**
Education	−0.007	−0.001 (0.001) (−0.002 to 0.001)	−0.57	0.57
STAI	0.024	0.002 (0.001) (−0.000 to 0.003)	1.64	0.10
GDS	−0.036	−0.004 (0.002) (−0.008 to −0.002)	−2.09	**0.04**
MMSE	−0.036	−0.006 (0.002) (−0.01 to −0.001)	−2.57	**0.01**
FH+	0.034	0.01 (0.01) (0.002 to 0.03)	2.35	**0.02**
*APOE* ε4	0.349	0.14 (0.01) (0.13 to 0.15)	24.97	**< 0.001**
CFI	0.105	0.01 (0.001) (0.007 to 0.01)	6.98	**< 0.001**
**Model 2: Covariates and AD concerns**				
Age	0.168	0.007 (0.001) (0.006 to 0.008)	11.73	**< 0.001**
Sex	−0.032	−0.01 (0.01) (−0.02 to −0.002)	−2.22	**0.03**
Education	−0.006	−0.000 (0.001) (−0.002 to 0.003)	−0.42	0.68
STAI	0.023	0.001 (0.001) (−0.000 to 0.003)	1.51	0.13
GDS	−0.035	−0.005 (0.002) (−0.007 to 0.000)	−2.22	**0.03**
MMSE	−0.035	−0.006 (0.002) (−0.01 to −0.001)	−2.46	**0.02**
FH+	0.026	0.01 (0.01) (−0.001 to 0.02)	1.72	0.08
*APOE* ε4	0.347	0.14 (0.01) (0.13 to 0.15)	24.78	**< 0.001**
CFI	0.098	0.009 (0.001) (0.006 to 0.01)	6.46	**< 0.001**
CADQ	0.038	0.002 (0.001) (0.001 to 0.003)	2.59	**0.01**
**Model 3: Covariates, AD concerns, and interactions**				
Age	0.168	0.007 (0.001) (0.006 to 0.008)	11.75	**< 0.001**
Sex	−0.077	−0.03 (0.03) (−0.08 to 0.02)	−1.22	0.22
Education	−0.007	−0.000 (0.001) (−0.002 to 0.001)	−0.46	0.64
STAI	0.023	0.001 (0.001) (−0.000 to 0.003)	1.53	0.13
GDS	−0.034	−0.005 (0.002) (−0.009 to −0.001)	−2.24	**0.03**
MMSE	−0.035	−0.006 (0.002) (−0.01 to −0.001)	−2.47	**0.01**
FH+	0.078	0.03 (0.03) (−0.02 to 0.08)	1.23	0.22
*APOE* ε4	0.209	0.08 (0.03) (0.03 to 0.14)	3.15	**0.002**
CFI	0.098	0.009 (0.001) (0.006 to 0.01)	6.45	**< 0.001**
CADQ	0.003	0.000 (0.002) (−0.004 to 0.004)	0.05	0.96
Sex * CADQ	0.052	0.001 (0.001) (−0.001 to 0.003)	0.73	0.47
FH+ * CADQ	−0.059	−0.001 (0.001) (−0.003 to 0.002)	−0.85	0.40
*APOE* ε4 * CADQ	0.144	0.003 (0.001) (0.000 to 0.005)	2.12	**0.03**

*Note*: Bold text indicates p values < 0.05 (i.e., considered statistically significant).

Abbreviations: *APOEε*4, apolipoprotein E4; CADQ, Concerns about developing Alzheimer's disease questionnaire; CFI, Cognitive Function Index; FH+, family history of dementia; GDS, Geriatric Depression Scale; MMSE, Mini‐Mental State Examination; STAI, State‐Trait Anxiety Inventory.

**FIGURE 2 alz70225-fig-0002:**
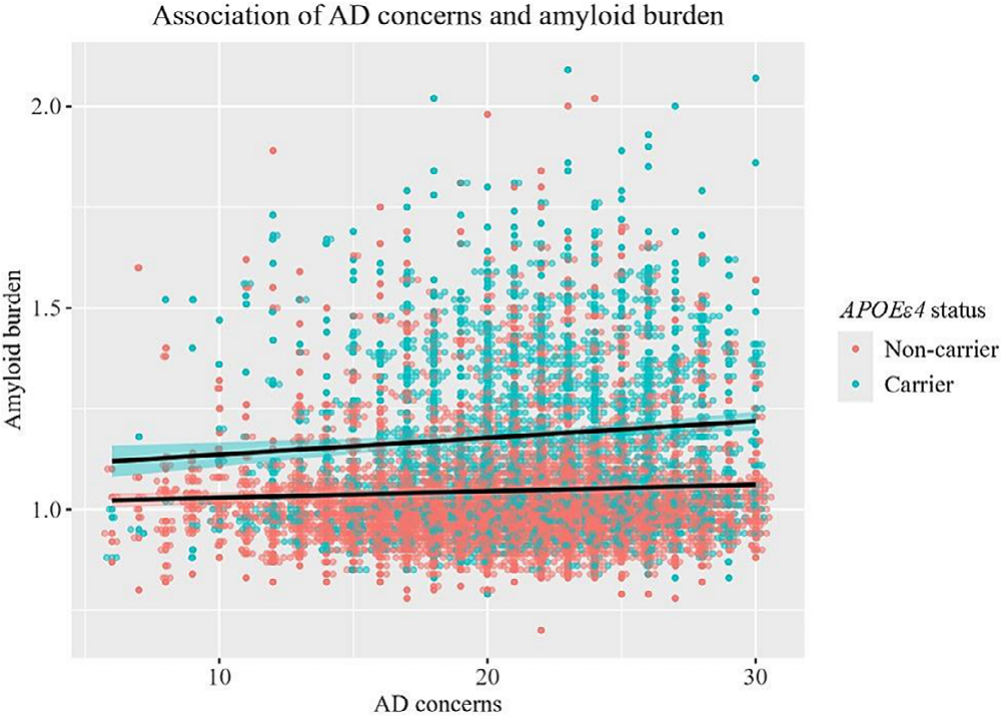
Scatterplot showing association of CADQ scores and amyloid burden by *APOE* ε4 status (carriers vs. non‐carriers). *APOE*, apolipoprotein E; CADQ, Concerns about developing Alzheimer's disease questionnaire.

In Model 3, there was a significant interaction between CADQ scores and *APOE* ε4 status; the association between CADQ scores and amyloid burden was stronger in individuals who were later found to be *APOE* ε4 carriers compared to non‐carriers (see Figure 2). To investigate this further, we computed separate regression models for *APOE* ε4 carriers and non‐carriers (see Table S3). Models confirmed a significant association between AD concerns and amyloid burden for *APOE* ε4 carriers, but not for non‐carriers. For *APOE* ε4 carriers, each 1‐unit increase of AD concern was associated with an increase in amyloid burden equivalent to ≈0.31 additional years of age (or 3.8 months). We then conducted separate analyses for *APOE* ε4 homozygotes and heterozygotes. The association between AD concerns and amyloid burden was significant for heterozygotes (*β* [95% CI] = 0.003 [0.000–0.005], *p *= 0.042) but not homozygotes (*β* [95% CI] = 0.000 [−0.009 to 0.009], *p *= 0.98).

There was no significant interaction of CADQ scores with sex or dementia family history. Comparison of Models 2 and 3 did not yield a significant difference (*F*(3,4439) = 1.79, *p* = 0.15). Models testing individual CADQ items are presented in Table S1. Three of the six items were significantly associated with elevated amyloid; the strongest association was observed for Item 2, “I am concerned that I will develop Alzheimer's disease dementia in the next 5 years.” Again, this association was stronger for *APOE* ε4 carriers compared to non‐carriers (see Table S4). Each 1‐unit increase in CADQ Item 2 was associated with an increase in amyloid burden equivalent to ≈0.53 additional years of age (or 6.4 months), compared to 0.29 additional years (3.4 months) in non‐carriers.

## 4 DISCUSSION

To our knowledge, this is the first study analyzing the association of AD concerns with amyloid burden in cognitively unimpaired individuals, adjusting for key demographics (age, sex, and education), anxiety and depressive symptoms, self‐reported and performance‐based cognitive performance, parental history of dementia, and genetic risk (*APOE* ε4). Our results[Fig alz70225-fig-0002] demonstrate that higher levels of concern about developing AD are associated with a core diagnostic biomarker for AD. Despite not knowing their genetic risk status, *APOE* ε4 carriers exhibited a stronger association between AD concerns and amyloid burden compared to non‐carriers. These findings suggest that self‐reported AD concern may be a useful risk indicator in cognitively unimpaired populations. This supports the assertion that people who are concerned about AD, particularly those who also carry genetic risk, are an important target population for early (i.e., pre‐clinical) intervention trials.

We compared the mean level of concern about developing AD across demographic, dementia risk, psychological, and lifestyle groups. Female participants reported higher AD concern compared to male participants, consistent with the previous literature.^7^ Sex and gender differences are well‐documented in AD risk and incidence.^23^, ^24^ Because women have a greater lifetime risk and provide most of the informal care for people living with AD and related dementias (ADRD), this higher level of concern could be expected. Similarly, we found that people who had a parent with dementia were more concerned about AD than those without a parental history of dementia. This supports the hypothesis that exposure, either as a family member or caregiver, is a determinant of AD worry.^7^ We did not find significant differences across racial groups, but AD concerns were higher among individuals who identified as Latino compared to those who identified as non‐Latino. This could reflect increased AD risk among Latino (compared to non‐Latino) populations^23^; however, it should be noted that most of our sample identified as non‐Latino (96%) and White (91%). Thus, the presence or absence of racial and ethnic group differences should be interpreted cautiously.

AD concerns were higher among participants with scores indicative of depression based on the GDS‐15 cutoff, in line with previous research.^7^, ^25^ Follow‐up analyses indicated that this association was specific to *APOE* ε4 carriers, consistent with evidence linking *APOE* ε4 to late‐life depression (independent of dementia).^26^, ^27^ Individuals with lower levels of AD concern more often met CDC guidelines for walking (>30 min per day). This association remained significant even after accounting for the effects of demographics and depressive symptoms. Individuals with lower levels of AD concern also more often met CDC guidelines for sleep (>7 h per night); however, this association was attenuated by demographics (sex, education) and anxiety and depression symptoms. Because our study was cross‐sectional, we cannot infer the causal direction of associations between AD concerns, mood, and lifestyle behaviors. It is possible that being more concerned about developing AD decreases motivation toward physical activity; however, the opposite could also be true, that is, lack of physical activity results in more AD concerns. Directionality notwithstanding, our findings demonstrate a significant association between AD concerns and modifiable lifestyle behaviors, as well as depression, which has public health implications for AD risk reduction.

Of interest, individuals with lower levels of AD concern did not meet CDC guidelines for aerobic exercise more often than those with higher AD concern. The reasons for the discrepancy in findings between daily walking and weekly exercise are unclear. However, walking outdoors has been reported to have beneficial effects above and beyond exercise alone, possibly by reducing rumination and enhancing positive emotions such as awe.^28^, ^29^ We also observed no significant group difference between current alcohol drinkers and non‐drinkers. This may be due to a lack of specificity in the measure used (i.e., a binary yes/no question), which did not capture type, quantity, or frequency of alcohol consumption.

Age, female sex, *APOE* ε4, depression, MMSE, and CFI scores were associated with higher amyloid burden. CFI score (a self‐report measure of cognitive complaints) was a particularly strong predictor across models, highlighting its utility as a screening tool. Including AD concerns as an additional predictor of amyloid burden in our model produced significantly better fit compared to the reference model with base predictors (demographics, mood, MMSE, CFI scores, family history, and genetic risk), suggesting that AD concerns have unique added value. It is important to note that the STAI score was not significantly predictive of amyloid burden, indicating that this was not an effect of general anxiety but rather an effect specific to the concerns of developing AD. The individual CADQ items that best predicted amyloid burden were the three items that explicitly referenced concerns (e.g., “My concern about developing Alzheimer's disease dementia is greater than my concern about other medical problems”). Items referencing beliefs (e.g., “I believe that I will someday develop Alzheimer's disease”) or preferences (e.g., “I would like to know if I am going to develop Alzheimer's disease”) were not significant.

There were also a number of significant interactions between AD concerns and *APOE* ε4 status. AD concerns were higher among participants who were later determined to be *APOE* ε4 carriers. It is possible that *APOE* ε4 carriers are aware of subtle cognitive changes that are not yet being identified on formal testing or questionnaires, including the CFI, which is then driving AD concerns. There was no significant interaction between AD concerns and parental family history of dementia on amyloid burden. This points to some discordance between *APOE* ε4 carriers and parental family history, which was reflected in our sample. Roughly half of *APOE* ε4 carriers (47.6%) had no parental history and vice versa. This could also reflect the study's strict criteria used for defining family history (i.e., diagnosis of dementia prior to age 80 years). The interaction with *APOE* but not dementia family history supports the hypothesis that other genetic or environmental risk factors are at play in the presence of the *APOE* ε4 allele,^30^, ^31^ which require further study. Gene–gene interactions are purported to be common in AD; for example, the apolipoprotein J (*APOJ*) gene influences amyloid aggregation and has been shown to interact with *APOE* ε4.^32^

Determining the meaning and clinical significance of AD concerns presents challenges. AD concerns are self‐reported and so may be vulnerable to biases like social desirability or fear of stigmatization. Indeed, AD is among the most feared and stigmatized age‐related conditions.^13^, ^33^ AD concerns can also be influenced by various personal, social, and cultural factors, making it difficult to establish standardized cutoffs for what constitutes high versus low. As with SCD, it remains unclear if there is a threshold for AD concerns that could be defined based on predictive value for subsequent cognitive decline.^34^ Another challenge is to define what constitutes “meaningful” individual‐level change in level of AD concerns over time.^35^ These challenges highlight the importance of evaluating AD concerns alongside clinical markers and biomarkers to better understand their significance.

AD is now being diagnosed with the aid of amyloid biomarkers and treated with anti‐amyloid therapies.^1^ Although biomarker testing is not recommended for asymptomatic populations outside of research and clinical trials,^36^ this is likely to change as new treatments emerge. Moreover, as has been highlighted recently, it may not be feasible to control access to these biomarkers when a diagnosis is made primarily on biomarker criteria.^2^ The current finding that AD concerns are associated with a core biological AD biomarker reinforces the importance of listening to individuals’ concerns, rather than dismissing them, as often occurs with those experiencing subjective complaints (i.e., unhelpfully termed the “worried well”). Going forward, screening more broadly for AD concerns could inform recruitment strategies for lifestyle interventions. Once identified, cognitively unimpaired individuals who are highly concerned about AD could be signposted to emerging brain health services for comprehensive risk profiling, communication, and supports.^37^

### 4.1 Limitations and future directions

Our study had limitations. The sample was highly educated, and the majority of participants were White non‐Latino, which limits generalizability to other populations. Relatedly, individuals enrolled in the A4 Study likely represent a highly motivated group, which is perhaps not fully representative of the general population because some individuals with high levels of AD concern may be less likely to participate in clinical research. Effective recruitment and retention strategies are therefore needed to engage diverse cognitively unimpaired populations going forward.^38^ Family history of dementia and lifestyle behaviors were self‐reported, which may be affected by recall bias. We focused on amyloid as a marker of AD pathology and did not assess associations with other core biomarkers, such as tau. We also focused on concerns about developing AD, which does not account for concerns about non‐AD dementias. Future studies should examine associations between dementia concerns and other core biomarkers. Future research could also investigate associations of concerns with amyloid positivity using various dichotomized thresholds (e.g., based on Centiloid scale cutoffs).

Measures of alcohol use and smoking lacked specificity. More detailed characterization of these behaviors (e.g., in terms of quantity, frequency, and reasons for engaging/abstaining) are needed to fully explore the association with AD concerns. Potential cofounders other than those accounted for in our models (e.g., socio‐economic status, income level, or other lifestyle factors) may have influenced our findings. The number of *APOE* ε4 homozygotes was relatively small, which limited our ability to investigate potential differences between homozygote and heterozygote sub‐groups. Given recent studies showing the effects of *APOE* ε4 homozygosity on AD pathology,^39^ further studies are needed to fully explore these differences. Finally, the study is cross‐sectional. Thus, we are unable to make causal inferences about AD concerns and pathological changes over time. Future studies are required with longitudinal data in more diverse samples to better understand the relationship of AD concerns with long‐term AD risk.

### 4.2 Conclusions

This study found that in cognitively unimpaired older adults, higher levels of concern about developing AD were associated with higher amyloid burden after adjusting for demographics, depression, cognitive performance, parental history of dementia, and *APOE* ε4 status. The finding that AD concerns are associated with a core AD biomarker in the preclinical stage has important clinical implications.

## CONFLICT OF INTEREST STATEMENT

The authors declare no conflicts of interest. Author disclosures are available in Supporting Information.

## CONSENT STATEMENT

All human subjects provided written informed consent.

## Supporting information



Supporting Information

Supporting Information
