# Supplementary material for: Association of Alzheimer's disease concerns with amyloid burden and lifestyle behaviors in cognitively unimpaired older adults
Source: Alzheimers Dement. 2025 Jun 6;21(6):e70225. doi: 10.1002/alz.70225 (PMC12142429; doi:10.1002/alz.70225)
Supplement: Supplementary file 2 — Supporting Information [file ALZ-21-e70225-s001.docx]

**Appendix 1**

| Table S1. Linear Regression Analyses for individual CADQ items | | | | | |  |
| --- | --- | --- | --- | --- | --- | --- |
| CADQ1: *I am concerned that I will develop Alzheimer's disease dementia* | | | | | |  |
|  | | **β** | **B (SE) [95% CI]** | ***t* Value** | ***P* value** |  |
| Age | | 0.172 | 0.007 (0.001) [0.006 to 0.008] | 11.97 | **<0.001** |  |
| Sex | | -0.03 | -0.01 (0.01) [-0.02 to -0.001] | -2.07 | **0.04** |  |
| Education | | -0.009 | -0.001 (0.001) [-0.003 to 0.001] | -0.65 | 0.52 |  |
| STAI | | 0.023 | 0.001 (0.001) [-0.000 to 0.003] | 1.52 | 0.13 |  |
| GDS | | -0.034 | -0.005 (0.002) [-0.008 to -0.001] | -2.19 | **0.03** |  |
| MMSE | | -0.035 | -0.006 (0.002) [-0.01 to -0.001] | -2.47 | **0.01** |  |
| FH+ | | 0.02 | 0.01 (0.01) [-0.004 to 0.02] | 1.36 | 0.18 |  |
| APOEε4 | | 0.345 | 0.14 (0.01) [0.13 to 0.15] | 24.68 | **<0.001** |  |
| CFI | | 0.098 | 0.009 (0.001) [0.006 to 0.01] | 6.46 | **<0.001** |  |
| CADQ1 | | 0.047 | 0.009 (0.003) [0.004 to 0.02] | 3.18 | **0.002** |  |
| CADQ2: *I am concerned that I will develop Alzheimer's disease dementia in the next 5 years* | | | | | | |
|  | | **β** | **B (SE) [95% CI]** | ***t* Value** | ***P* value** |  |
| Age | | 0.161 | 0.007 (0.001) [0.006 to 0.008] | 11.16 | **<0.001** |  |
| Sex | | -0.031 | -0.01 (0.01) [-0.02 to -0.001] | -2.19 | **0.03** |  |
| Education | | -0.005 | -0.000 (0.001) [-0.002 to 0.002] | -0.37 | 0.71 |  |
| STAI | | 0.021 | 0.001 (0.001) [-0.001 to 0.003] | 1.40 | 0.16 |  |
| GDS | | -0.036 | -0.005 (0.002) [-0.009 to -0.001] | -2.33 | **0.02** |  |
| MMSE | | -0.033 | -0.005 (0.002) [-0.01 to -0.001] | -2.33 | **0.02** |  |
| FH+ | | 0.024 | 0.01 (0.01) [-0.001 to 0.02] | 1.68 | 0.09 |  |
| APOEε4 | | 0.345 | 0.14 (0.01) [0.13 to 0.15] | 24.71 | **<0.001** |  |
| CFI | | 0.092 | 0.009 (0.001) [0.006 to 0.01] | 6.07 | <0.001 |  |
| CADQ2 | | 0.062 | 0.01 (0.002) [0.006 to 0.02] | 4.24 | <0.001 |  |
| CADQ3: *I would like to know if I am going to develop Alzheimer's disease dementia at some point later in my life* | | | | | | |
|  | | **β** | | **B (SE) [95% CI]** | ***t* Value** | ***P* value** |
| Age | | 0.168 | | 0.007 (0.001) [0.006 to 0.008] | 11.69 | **<0.001** |
| Sex | | -0.034 | | -0.01 (0.01) [-0.02 to -0.002] | -2.38 | **0.02** |
| Education | | -0.009 | | -0.001 (0.001) [-0.003 to 0.001] | -0.62 | 0.53 |
| STAI | | 0.024 | | 0.001 (0.001) [-0.000 to 0.003] | 1.62 | 0.11 |
| GDS | | -0.033 | | -0.004 (0.002) [-0.008 to -0.000] | -2.09 | **0.04** |
| MMSE | | -0.036 | | -0.006 (0.002) [-0.01 to -0.001] | -2.51 | **0.01** |
| FH+ | | 0.033 | | 0.01 (0.01) [0.002 to 0.02] | 2.33 | **0.02** |
| APOEε4 | | 0.348 | | 0.14 (0.01) [0.13 to 0.15] | 24.90 | **<0.001** |
| CFI | | 0.105 | | 0.009 (0.001) [0.006 to 0.01] | 7.01 | **<0.001** |
| CADQ3 | | -0.002 | | -0.001 (0.003) [-0.006 to 0.005] | -0.17 | 0.86 |
| CADQ4: *I believe that I will someday develop Alzheimer's disease dementia* | | | | | | |
|  | | **β** | | **B (SE) [95% CI]** | ***t* Value** | ***P* value** |
| Age | | 0.168 | | 0.007 (0.001) [0.006 to 0.008] | 11.72 | **<0.001** |
| Sex | | -0.034 | | -0.01 (0.01) [-0.02 to -0.002] | -2.36 | **0.02** |
| Education | | -0.008 | | -0.001 (0.001) [-0.002 to 0.001] | -0.59 | 0.55 |
| STAI | | 0.024 | | 0.001 (0.001) [-0.000 to 0.003] | 1.61 | 0.11 |
| GDS | | -0.033 | | -0.004 (0.002) [-0.008 to -0.000] | -2.11 | **0.04** |
| MMSE | | -0.035 | | -0.006 (0.002) [-0.01 to -0.001] | -2.49 | **0.01** |
| FH+ | | 0.031 | | 0.01 (0.01) [0.001 to 0.02] | 2.11 | **0.04** |
| APOEε4 | | 0.348 | | 0.14 (0.01) [0.13 to 0.15] | 24.84 | **<0.001** |
| CFI | | 0.104 | | 0.01 (0.001) [0.007 to 0.01] | 6.82 | **<0.001** |
| CADQ4 | | 0.008 | | 0.001 (0.003) [-0.004 to 0.007] | 0.53 | 0.60 |
| CADQ5: *Alzheimer's disease dementia is the worst disease I can think of* | | | | | | |
|  | | **β** | | **B (SE) [95% CI]** | ***t* Value** | ***P* value** |
| Age | | 0.168 | | 0.007 (0.001) [0.006 to 0.008] | 11.69 | **<0.001** |
| Sex | | -0.033 | | -0.01 (0.01) [-0.02 to -0.002] | -2.31 | **0.02** |
| Education | | -0.008 | | -0.001 (0.001) [-0.002 to 0.001] | -0.53 | 0.59 |
| STAI | | 0.024 | | 0.001 (0.001) [-0.000 to 0.003] | 1.60 | 0.11 |
| GDS | | -0.033 | | -0.004 (0.002) [-0.008 to -0.000] | -2.10 | **0.04** |
| MMSE | | -0.034 | | -0.006 (0.002) [-0.01 to -0.001] | -2.43 | **0.02** |
| FH+ | | 0.033 | | 0.01 (0.01) [-0.002 to 0.02] | 2.31 | **0.02** |
| APOEε4 | | 0.348 | | 0.14 (0.01) [0.13 to 0.15] | 24.90 | **<0.001** |
| CFI | | 0.104 | | 0.01 (0.001) [0.007 to 0.01] | 6.97 | **<0.001** |
| CADQ5 | | 0.016 | | 0.002 (0.002) [0.002 to 0.006] | 1.17 | 0.24 |
| CADQ6: *My concern about developing Alzheimer's disease dementia is greater than my concern about other medical problems* | | | | | | |
|  | | **β** | | **B (SE) [95% CI]** | ***t* Value** | ***P* value** |
| Age | | 0.169 | | 0.007 (0.001) [0.006 to 0.008] | 11.80 | **<0.001** |
| Sex | | -0.032 | | -0.01 (0.01) [-0.02 to -0.001] | -2.27 | **0.02** |
| Education | | -0.008 | | -0.001 (0.001) [-0.003 to 0.001] | -0.59 | 0.55 |
| STAI | | 0.023 | | 0.001 (0.001) [-0.000 to 0.003] | 1.53 | 0.13 |
| GDS | | -0.034 | | -0.004 (0.002) [-0.008 to -0.001] | -2.17 | **0.03** |
| MMSE | | -0.035 | | -0.006 (0.002) [-0.01 to -0.001] | -2.44 | **0.02** |
| FH+ | | 0.028 | | 0.01 (0.01) [0.000 to 0.02] | 1.96 | 0.05 |
| APOEε4 | | 0.347 | | 0.14 (0.01) [0.13 to 0.15] | 24.81 | **<0.001** |
| CFI | | 0.102 | | 0.009 (0.001) [0.006 to 0.01] | 6.75 | **<0.001** |
| CADQ6 | | 0.031 | | 0.005 (0.002) [0.005 to 0.008] | 2.20 | **0.03** |

Abbreviations: APOE, apolipoprotein E; CADQ, Concerns about developing Alzheimer’s disease Questionnaire; CFI, Cognitive Function Index; FH+, family history of dementia; GDS, Geriatric Depression Scale; MMSE, Mini-Mental State Examination Score; STAI, State-Trait Anxiety Inventory.

| Table S2. Depression and Lifestyle Factor Logistic Regression Analyses | | | | |
| --- | --- | --- | --- | --- |
| Depressive symptoms (‘normal’ vs. ‘depression’) | | | | |
|  | **B (SE) [95% CI]** | ***z* Value** | | ***P* value** |
| Age | 0.033 (0.018) [-0.004 to 0.068] | 1.758 | | 0.08 |
| Sex | 0.167 (0.19) [-0.209 to 0.536] | 0.879 | | 0.38 |
| Education | -0.072 (0.032) [-0.137 to -0.009] | -2.23 | | **0.03** |
| STAI | 0.319 (0.024) [0.271 to 0.367] | 13.088 | | **<0.001** |
| APOEε4 | -2.245 (1.062) [-4.401 to -0.225] | -2.114 | | **0.04** |
| CADQ | 0.028 (0.023) [-0.018 to 0.075] | 1.185 | | 0.24 |
| APOEε4 * CADQ | 0.091 (0.045) [0.004 to 0.18] | 2.019 | | **0.04** |
| Daily walking (≥30 minutes vs. <30 minutes) | | | | |
|  | **B (SE) [95% CI]** | ***z* Value** | ***P* value** | |
| Age | -0.026 (0.007) [-0.04 to -0.011] | -3.5 | **<0.001** | |
| Sex | -0.194 (0.072) [-0.335 to -0.052] | -2.68 | **0.007** | |
| Education | 0.005 (0.012) [-0.019 to 0.03] | 0.43 | 0.671 | |
| STAI | -0.009 (0.012) [-0.033 to 0.014] | -0.78 | 0.434 | |
| GDS | -0.093 (0.024) [-0.141 to -0.045] | -3.84 | **<0.001** | |
| APOEε4 | -0.238 (0.35) [-0.922 to 0.452] | -0.68 | 0.497 | |
| CADQ | -0.019 (0.009) [-0.038 to -0.001] | -2.07 | **0.039** | |
| APOEε4 * CADQ | 0.013 (0.016) [-0.018 to 0.045] | 0.83 | 0.404 | |
| Nightly sleep (≥7 hours minutes vs. <7 hours) | | | | |
|  | **B (SE) [95% CI]** | ***z* Value** | ***P* value** | |
| Age | -0.005 (0.007) [-0.02 to 0.009] | -0.74 | 0.459 | |
| Sex | -0.14 (0.071) [-0.28 to 0] | -1.97 | **0.049** | |
| Education | 0.051 (0.012) [0.027 to 0.075] | 4.11 | **<0.001** | |
| STAI | -0.056 (0.012) [-0.079 to -0.034] | -4.88 | **<0.001** | |
| GDS | -0.087 (0.024) [-0.133 to -0.04] | -3.66 | **<0.001** | |
| APOEε4 | 0.049 (0.342) [-0.619 to 0.724] | 0.14 | 0.886 | |
| CADQ | -0.004 (0.009) [-0.022 to 0.014] | -0.42 | 0.672 | |
| APOEε4 * CADQ | -0.003 (0.016) [-0.034 to 0.028] | -0.19 | 0.846 | |
| Alcohol use (yes vs. no) | | | | |
|  | **B (SE) [95% CI]** | ***z* Value** | ***P* value** | |
| Age | -0.000 (0.007) [-0.013 to 0.013] | -0.06 | 0.950 | |
| Sex | 0.398 (0.063) [0.274 to 0.522] | 6.28 | **<0.001** | |
| Education | 0.038 (0.011) [0.017 to 0.060] | 3.53 | **0.004** | |
| STAI | -0.012 (0.011) [-0.009 to 0.032] | 1.12 | 0.264 | |
| GDS | -0.088 (0.022) [-0.133 to -0.044] | -3.86 | **0.000** | |
| APOEε4 | -0.595 (0.303) [-1.189 to -0.001] | -1.96 | 0.050 | |
| CADQ | -0.008 (0.008) [-0.024 to 0.008] | -0.92 | 0.356 | |
| APOEε4 * CADQ | 0.031 (0.014) [0.004 to 0.058] | 2.22 | **0.026** | |
| Smoking (yes vs. no) | | | | |
|  | **B (SE) [95% CI]** | ***z* Value** | ***P* value** | |
| Age | -0.047 (0.027) [-0.102 to 0.006] | -1.70 | 0.089 | |
| Sex | 0.560 (0.241) [0.088 to 1.034] | 2.33 | 0.020 | |
| Education | -0.050 (0.042) [-0.130 to 0.037] | -1.08 | 0.281 | |
| STAI | -0.015 (0.040) [-0.096 to 0.062] | -0.38 | 0.707 | |
| GDS | 0.220 (0.062) [0.092 to 0.338] | 3.54 | **<0.001** | |
| APOEε4 | 0.346 (1.146) [-1.947 to 2.561] | 0.30 | 0.763 | |
| CADQ | 0.006 (0.033) [-0.058 to 0.073] | 0.17 | 0.862 | |
| APOEε4 * CADQ | -0.003 (0.052) [-0.105 to 0.101] | -0.05 | 0.959 | |
| Weekly aerobic exercise (≥2.5 hours vs. <2.5 hours) | | | | |
|  | **B (SE) [95% CI]** | ***z* Value** | ***P* value** | |
| Age | -0.021 (0.007) [-0.034 to -0.008] | -3.19 | **0.002** | |
| Sex | 0.273 (0.064) [0.148 to 0.399] | 4.27 | **<0.001** | |
| Education | 0.092 (0.011) [0.070 to 0.114] | 8.17 | **<0.001** | |
| STAI | 0.009 (0.011) [-0.012 to 0.029] | 0.80 | 0.421 | |
| GDS | -0.168 (0.024) [-0.216 to -0.122] | -7.06 | **<0.001** | |
| APOEε4 | 0.091 (0.306) [-0.509 to 0.691] | 0.30 | 0.766 | |
| CADQ | 0.009 (0.009) [-0.008 to 0.025] | 1.08 | 0.281 | |
| APOEε4 * CADQ | -0.004 (0.014) [-0.031 to 0.024] | -0.27 | 0.791 | |

Abbreviations: APOE, apolipoprotein E; CADQ, Concerns about developing Alzheimer’s disease Questionnaire; CFI, Cognitive Function Index; FH+, family history of dementia; GDS, Geriatric Depression Scale; MMSE, Mini-Mental State Examination Score; STAI, State-Trait Anxiety Inventory.

| Table S3. Amyloid Burden Linear Regression Analyses by APOEε4 status | | | | |
| --- | --- | --- | --- | --- |
|  | **β** | **B (SE) [95% CI]** | ***t* Value** | ***P* value** |
| APOEε4 carriers |  |  |  |  |
| Age | 0.189 | 0.801 (0.176) [0.455 to 1.147] | 4.54 | **<0.001** |
| Sex | -0.008 | 0.01 (0.001) [0.007 to 0.012] | 7.34 | **<0.001** |
| Education | -0.020 | -0.004 (0.01) [-0.026 to 0.019] | -0.33 | 0.742 |
| STAI | 0.000 | -0.002 (0.002) [-0.006 to 0.002] | -0.78 | 0.435 |
| GDS | -0.021 | 0 (0.002) [-0.004 to 0.004] | 0.01 | 0.989 |
| MMSE | -0.068 | -0.003 (0.004) [-0.011 to 0.005] | -0.74 | 0.463 |
| FH+ | 0.021 | -0.012 (0.005) [-0.022 to -0.003] | -2.67 | **0.008** |
| CFI | 0.131 | 0.013 (0.003) [0.008 to 0.018] | 4.76 | **<0.001** |
| CADQ | 0.059 | 0.003 (0.002) [0.000 to 0.005] | 2.31 | **0.021** |
| APOEε4 non-carriers | | | | |
| Age | 0.178 | 0.006 (0.001) [0.005 to 0.007] | 7.410 | **<0.001** |
| Sex | -0.051 | -0.016 (0.006) [-0.028 to -0.004] | 9.420 | **<0.001** |
| Education | 0.002 | 0.000 (0.001) [-0.002 to 0.002] | -2.690 | **0.007** |
| STAI | 0.044 | 0.002 (0.001) [0 to 0.004] | 0.090 | 0.930 |
| GDS | -0.052 | -0.006 (0.002) [-0.01 to -0.001] | 2.180 | **0.029** |
| MMSE | -0.010 | -0.001 (0.002) [-0.006 to 0.003] | -2.530 | **0.011** |
| FH+ | 0.031 | 0.010 (0.006) [-0.002 to 0.021] | -0.550 | 0.582 |
| CFI | 0.080 | 0.001 (0.001) [0 to 0.002] | 3.990 | **<0.001** |
| CADQ | 0.031 | 0.006 (0.001) [0.005 to 0.007] | 1.550 | 0.122 |

Abbreviations: APOE, apolipoprotein E; CADQ, Concerns about developing Alzheimer’s disease Questionnaire; CFI, Cognitive Function Index; FH+, family history of dementia; GDS, Geriatric Depression Scale; MMSE, Mini-Mental State Examination Score; STAI, State-Trait Anxiety Inventory.

| Table S4. CADQ2 Regression Analyses for APOEε4 carriers vs. non-carriers | | | | |
| --- | --- | --- | --- | --- |
|  | **β** | **B (SE) [95% CI]** | ***t* Value** | ***P* value** |
| APOEε4 carriers |  |  |  |  |
| CADQ2 | 0.095 | 0.018 (0.005) [0.009 to 0.03] | 3.71 | **<0.001** |
| APOEε4 non-carriers | | | | |
| CADQ2 | 0.049 | 0.006 (0.003) [0.001 to 0.01] | 2.53 | **0.01** |

Abbreviations: APOE, apolipoprotein E; CADQ2, Concerns about developing Alzheimer’s disease Questionnaire Item 2, “I am concerned that I will develop Alzheimer's disease dementia in the next 5 years”.
